# A resource for understanding and evaluating outcomes of undergraduate field experiences

**DOI:** 10.1002/ece3.8241

**Published:** 2021-11-19

**Authors:** Erin E. Shortlidge, Alison Jolley, Stephanie Shaulskiy, Emily Geraghty Ward, Christopher N. Lorentz, Kari O'Connell

**Affiliations:** ^1^ Department of Biology Portland State University Portland Oregon USA; ^2^ Te Puna Ako – Centre for Tertiary Teaching & Learning University of Waikato Hamilton New Zealand; ^3^ Biological Station University of Michigan Ann Arbor Michigan USA; ^4^ Geology Program Rocky Mountain College Billings Montana USA; ^5^ Department of Biological Sciences Thomas More University Crestview Hills Kentucky USA; ^6^ STEM Research Center Oregon State University Corvallis Oregon USA

**Keywords:** assessment, field experiences, inclusion, learning outcomes, undergraduates

## Abstract

Undergraduate field experiences (UFEs) are a prominent element of science education across many disciplines; however, empirical data regarding the outcomes are often limited. UFEs are unique in that they typically take place in a field setting, are often interdisciplinary, and include diverse students. UFEs range from courses, to field trips, to residential research experiences, and thereby have the potential to yield a plethora of outcomes for undergraduate participants. The UFE community has expressed interest in better understanding how to assess the outcomes of UFEs. In response, we developed a guide for practitioners to use when assessing their UFE that promotes an evidence‐based, systematic, iterative approach. This essay guides practitioners through the steps of: identifying intended UFE outcomes, considering contextual factors, determining an assessment approach, and using the information gained to inform next steps. We provide a table of common learning outcomes with aligned assessment tools, and vignettes to illustrate using the assessment guide. We aim to support comprehensive, informed assessment of UFEs, thus leading to more inclusive and reflective UFE design, and ultimately improved student outcomes. We urge practitioners to move toward evidence‐based advocacy for continued support of UFEs.

## Graphical abstract

This paper serves as a guide to best practices in assessment and evaluation of undergraduate field experiences. We offer a response to field educators who want to learn how to effectively evaluate outcomes from field learning experiences.

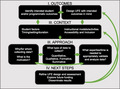

## 1 INTRODUCTION

### 1.1 Background

Conducting research, collecting data, and teaching students outside of a laboratory or classroom setting are commonplace across disciplines. For many scientists, being “in the field” is paramount to the work that they do (Cutter, 1993; Rudwick, 1996; Wilson, 1982). Therefore, in numerous disciplines, engaging undergraduates in experiences that take place in the field ais not only expected and intuitive (Dressen, 2002), but also considered central to training goals (Fleischner et al., 2017; Giles et al., 2020; Gold et al., 1994). For the purposes of this paper, we borrow from the work of colleagues (Fleischner et al., 2017; Morales et al., 2020; O’Connell et al., 2021) to define what we are considering to be a UFE. UFEs are designed explicitly with student learning in mind and occur in a field setting where students engage with the natural world, or through a virtual experience, meant to mimic an experience in the field. UFEs can take place in a variety of settings and durations including immersive, residential courses or programs at field stations and marine laboratories, short field trips as part of traditional on‐campus university courses, or long, multi‐day field trips. The COVID‐19 pandemic has further encouraged the development of remote UFEs and challenged us to reflect on how lessons in field education design might apply beyond in‐person settings (e.g., Barton, 2020). The discussion that follows largely applies to in‐person as well as remote UFEs. Further, we are not limiting our discussion of UFEs to a few prominent disciplines, as we are aware of the wide‐range of UFEs, and aim to be inclusive.

Some have argued that a student's undergraduate experience in disciplines such as biology, ecology, and the geosciences is not complete without a UFE (Cutter, 1993; Klemow et al., 2019; Nairn, 1999; Petcovic et al., 2014). A survey of participants at the Geological Society of America meetings (2010 and 2011) showed that the majority (89%) of survey participants felt that field experiences were vital to geoscience education and that the bulk of the value lies in cognitive gains, and to a lesser degree, sustained interest in the field (Petcovic et al., 2014). The Governing Board of the Ecological Society of America showed strong support of UFEs by including fieldwork and the ability to apply natural history approaches as two of the ecology practices in the recently adopted Four‐Dimensional Ecology Education Framework (Klemow et al., 2019).

Participating in a UFE can spark students’ interest in the scientific topic being explored in the field (Dayton & Sala, 2001; LaDue & Pacheco, 2013; Petcovic et al., 2014), increase student cognitive gains in disciplinary content (Easton & Gilburn, 2012; Scott et al., 2012), improve student understanding of the process of science (Patrick, 2010), foster development of discipline‐specific technical skills (Peasland et al., 2019), and increase persistence in STEM fields (Jelks & Crain, 2020). UFEs can also have far‐reaching impacts, even changing the trajectory of students’ lives by influencing career choices, or solidifying long‐term commitments to the environment (Barker et al., 2002; Palmer & Suggate, 1996). UFEs have been identified as critical contributors to students’ development of a sense of place (Billick & Price, 2010; Jolley, Kennedy, et al., 2018; Semken, 2005; Semken et al., 2017; Van Der Hoeven Kraft et al., 2011) as well as fostering a resonance with Indigenous peoples and Traditional Ecological Knowledge (Cajete, 2000; Riggs, 2005).

Despite these key outcomes, some have voiced fears about field experiences going “extinct” and have sounded alarm bells for stakeholders to consider how to gain further support for such experiences (Barker et al., 2002; Swing et al., 2021; Whitmeyer et al., 2009a). There is a widespread occurrence of, and in many cases, fervent advocacy for undergraduates learning in the field. Yet, there is a lack of systematically collected data on specific outcomes resulting from the diversity of possible field experiences (Mogk & Goodwin, 2012). Practitioners (field instructors, directors, coordinators, and staff) want to understand the efficacy of their individual programs, while universities and funding agencies require evidence of success for continued support of undergraduate field programs. Stakeholders across disciplines have made it clear that more empirical studies that test claims of positive student outcomes are needed for continued support of UFEs (Clift & Brady, 2005; NRC, 2014; O'Connell et al., 2018; Smith, 2004). This is particularly true as it relates to improving equity, access, and inclusion in the field (NRC, 2003, Brewer & Smith, 2011; Wieman, 2012; Morales et al., 2020). Collecting evidence of student outcomes will help to identify opportunities and challenges for supporting the inclusion of all students in UFEs and aid in tackling some of the challenges with inclusion that we already know exist (O’Connell et al., 2021).

Practitioners report an interest in collecting evidence of outcomes from their UFEs for iterative improvement, to demonstrate value of their programs, and to contribute to broader understanding of field learning, but do not feel confident in their ability to measure student outcomes, given that it is not their expertise (O’Connell et al., 2020). Indeed, most of the studies that have measured outcomes from UFEs are conducted by education researchers, trained in quantitative and/or qualitative research methods. To meet practitioners where they are, and support mindful, efficacious assessment of UFEs, we: (1) present a resource for practitioners to use when they want to assess UFE outcomes and improve their programs and courses, (2) address how assessment and evaluation of UFE outcomes can help practitioners better design inclusive field experiences, and (3) identify an existing pool of instruments that align with intended student outcomes of UFEs.

### 1.2 Conceptualization of this paper

The authors of this paper are members and founders of the Undergraduate Field Experiences Research Network (UFERN; www.ufern.net), a NSF‐funded Research Coordination Network focused on fostering effective UFEs. UFERN brings together diverse perspectives and expertise to examine the potentially distinctive learning and personal growth that happens for students when they engage in UFEs across the range of disciplines and formats. During a UFERN meeting (2019), it became apparent that undergraduate field educators from across disciplines were frequently requesting help in how to collect empirical evidence about complex student outcomes from UFEs (O’Connell et al., 2020). The work presented here emerged from conversations at that UFERN meeting and is a collaboration between STEM education researchers, social scientists, and undergraduate field educators from multiple disciplines, to directly address calls for guidance on assessing UFEs.

### 1.3 Strategies for assessing UFEs

**FIGURE 1:**
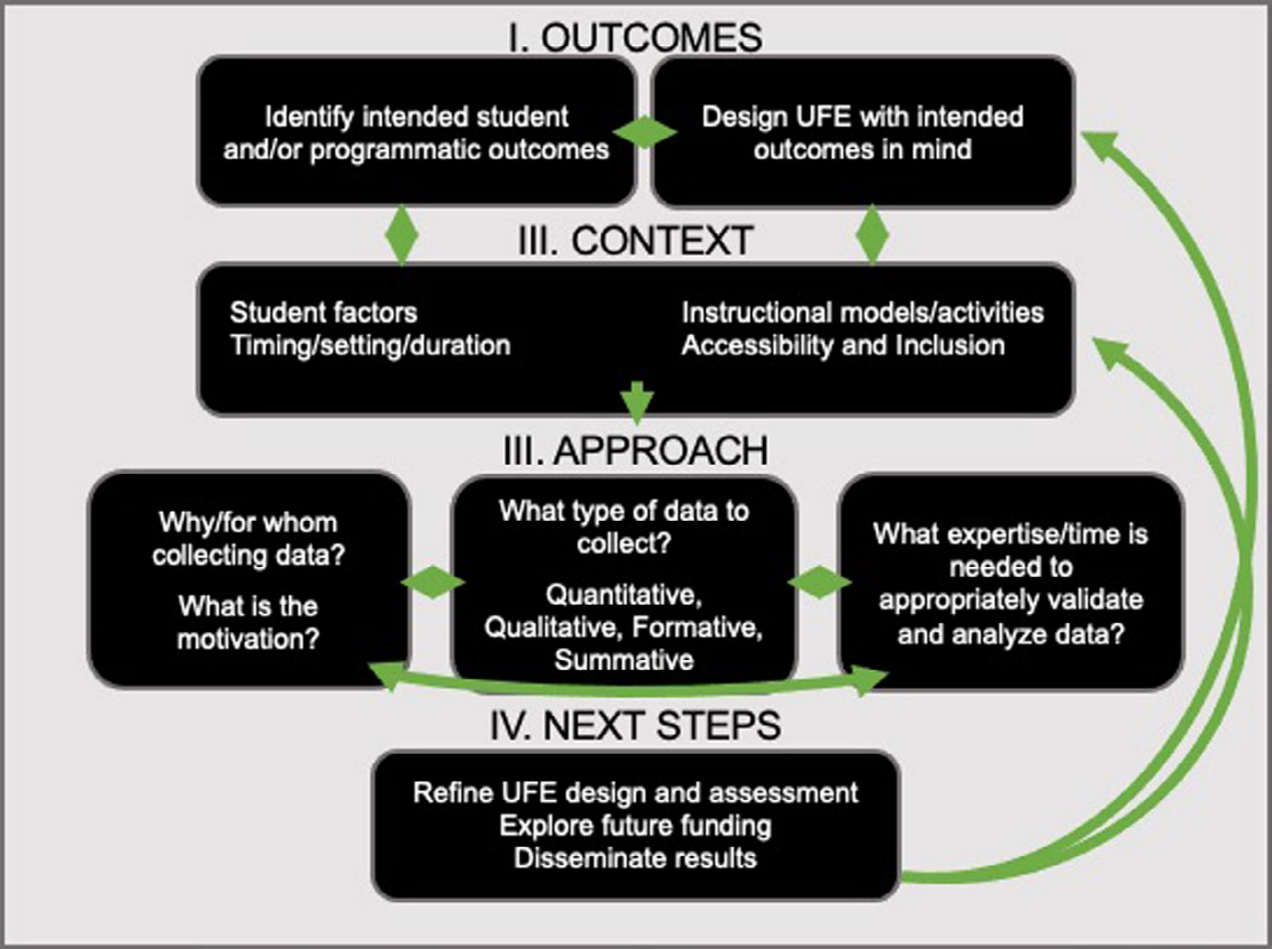
Guide for Assessing Undergraduate Field Experiences (UFEs). The figure presents a guide to walk practitioners through assessing their UFE. The green arrows signify that each box informs the other, and iterative reflection and refinement are a key aspect of informed evaluation and assessment

We advocate that stakeholders work to understand and evaluate their UFEs or UFE programs in clear alignment with the unique goals of each individual field experience. Reflecting best practices in designing learning environments that support student gains, we draw from the process described as “backwards design” (Wiggins et al., 1998). Importantly, this method emphasizes the alignment of UFE design to the outcomes being measured. We build from a “how to” guide designed for assessing course‐based undergraduate research experiences (CUREs) presented by Shortlidge and Brownell (2016) and have expanded and tailored the guide to be specific to UFEs. Figure 1 is to be used as a guide and a mechanism for reflection, allowing practitioners to refine a UFE to better serve the students, meet the intended outcomes, and/or change and build upon data collection methods already in place.

We provide guide that is inclusive to those who intend to assess, evaluate, and/or conduct education research on UFEs, and therefore will describe how these are separate but interrelated and likely overlapping actions. In order to clarify potential misunderstandings, we explain the language that we use regarding assessment, evaluation, and research.

We use the word *assessment* when we are referring to measuring student learning outcomes from UFEs. Assessment tools refer to the instruments that are used to collect the outcome data (e.g., a survey, rubric, or essay). Assessment may be qualitative (e.g., interviews), quantitative (e.g., surveys), or a mix of approaches (Creswell, 2013).

A *programmatic evaluation* might aim to holistically understand the experience that all or individual stakeholders have in a UFE; the evaluation could include students, instructors, program directors, and community partners. To evaluate something is to determine its merit, value, or significance (Patton, 2008), and program evaluation has been described as “the systematic assessment of the operation and/or outcomes of a program or policy, compared to a set of explicit or implicit standards as a means of contributing to the improvement of the program or policy” (Shackman, 2008). Thus, an evaluation of a UFE would determine the appropriate assessment methodology and identify whether programmatic goals are being met. Such information can inform how a UFE can be improved. Evaluation is often conducted by an external evaluator who may work with the UFE leadership team to develop a plan, often through the creation and use of a site‐specific logic model (Taylor‐Powell & Henert, 2008). An evaluation may target a range of UFEs, from a singular disciplinary program, or an entire field station's season of hosted UFEs.

The collection of empirical evidence about a UFE, which can be gathered through assessment and evaluation, and adds new knowledge, could potentially be used for education *research*. The authors Towne & Shavelson state that: “…education research serves two related purposes: to add to fundamental understanding of education‐related phenomena and events, and to inform practical decision making… both require researchers to have a keen understanding of educational practice and policy, and both can ultimately lead to improvements in practice.” (Towne & Shavelson, 2002, p. 83).

If the aim is to publish research outcomes from a UFE, practitioners will likely need to submit a proposal to an Institutional Review Board (IRB). The IRB can then determine whether a human subjects’ research exemption or expedition protocol will be necessary. If an IRB protocol is needed, this should occur *before* data collection begins. Gaining IRB approval is contingent on researchers having been certified in human subjects’ research and a robust and detailed research plan that follows human subjects’ research guidelines. Thus, conducting education research on UFEs requires advance planning and ideally would be conducted in partnership with or with advisement from education researchers. Typically, if a study is IRB approved, participants of the study need to consent to their information to be used for research purposes.

Publishing outcomes may be desirable, but not all data will be collected in a way that yields publishable results, yet those results may be highly informative to practitioners and UFE programs. Designing effective formative assessments to understand and modify a UFE might be the most appropriate workflow before engaging in intentional research studies on the outcomes of a UFE. Importantly, we do not advocate that one method is better, or more or less appropriate than another; the approach should depend on the aims and intentions of the stakeholders and the resources available.

### 1.4 Guide to assessing UFEs and sample vignettes

Figure 1 is presented as a guide for practitioners to use for understanding the outcomes of a UFE. The green arrows signify that each box informs the other, and iterative reflection and refinement are a key aspect of informed evaluation and assessment. The guide includes four key components: (I) identifying the intended student and/or programmatic *outcomes* for the UFE; (II) considering the *context* of the UFE, which may include any number of factors related to: setting, duration, timing, discipline, student identity, and accessibility of the UFE; (III) defining an assessment *approach* that is appropriate for the context and in alignment with the intended outcomes; and (IV) utilizing the outcomes and approach to inform and refine *next steps* in the UFE.

**FIGURE 2:**
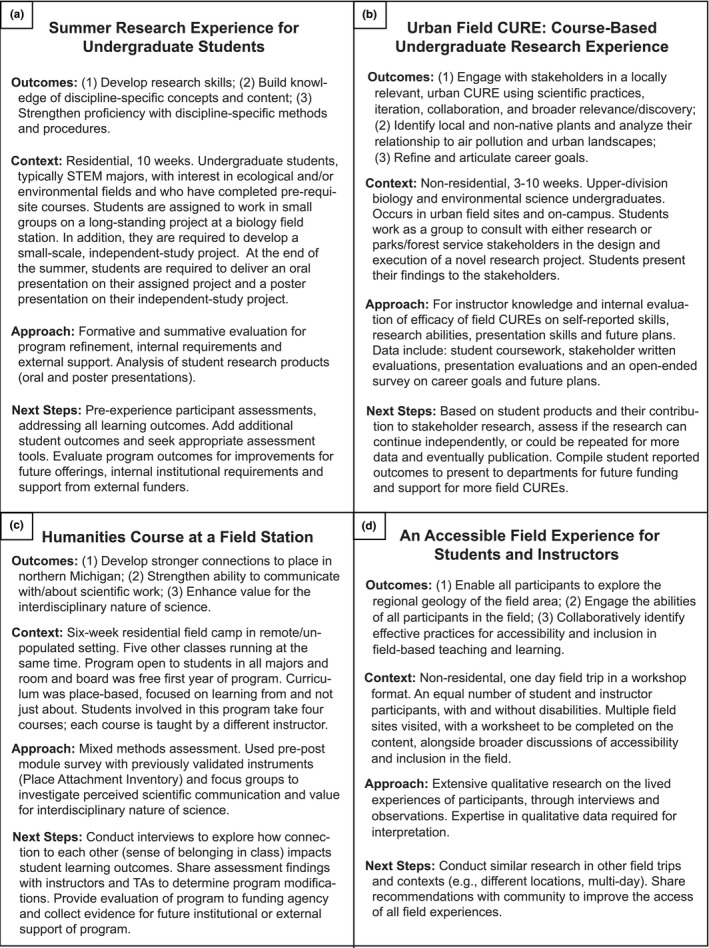
Vignettes of Undergraduate Field Experiences (UFEs). These vignettes (a–d) represent actual examples of UFEs and illustrate how to apply the components of Figure 1 (Strategy for Assessment of Undergraduate Field Experiences (UFEs)) to assess each UFE. Figure 2d was based on (Feig et al., 2019; Gilley et al., 2015; Stokes et al., 2019)

To highlight diverse UFEs and give realistic examples of assessment and evaluation approaches, we present four examples of UFEs, referred to as “vignettes” (Figure 2). The vignettes provide examples of how one can apply the components of the guide (Figure 1) to a given UFE, and at the end of the paper, we present two of the vignettes in a more detailed narrative, offering examples that synthesize the ideas presented (*Expanded Vignettes*).

#### 1.4.1 Identify the intended outcomes from the UFE

The main focus of this work is to provide the tools and resources needed such that stakeholders can confidently assess whether students are meeting expected learning outcomes from UFEs. Such learning outcomes could be: students expand their knowledge of endemic amphibians, or students report an increased interest in environmental sustainability. Programmatic outcomes and goals (e.g., participants are involved in community engagement and scientific knowledge‐building activities) are also critical components of this type of learning environment, and thus are also represented in example vignettes (Figure 2).

We draw upon Bloom's Taxonomy of Learning (Anderson et al., 2001; Bloom & Krathwohl, 1966) to aid practitioners in considering the possible outcomes from UFEs. The taxonomy describes three fundamental domains of learning: the *cognitive, affective*, and *psychomotor* domains. Studies about UFEs demonstrate that students may experience outcomes across all of these domains and more (Boyle et al., 2007; O’Connell et al., 2020; Petcovic et al., 2014; Scott et al., 2012, 2019; Stokes & Boyle, 2009). *Cognitive* outcomes from a UFE could include: an improved ability to explain plant species interactions, accurately identify geological formations, or solve a problem using an interdisciplinary lens (Bauerle & Park, 2012; Fuller et al., 2006; Tripp et al., 2020). *Affective* outcomes could include: a newfound interest in a subject, such as conservation; motivation to continue seeking out field learning experiences; or, development of a connection to place (Boyle et al., 2007; Jolley, Kennedy, et al., 2018; Scott et al., 2019; Simm & Marvell, 2015). Outcomes in the *psychomotor* domain could include: the improved ability to geolocate, collect and measure sediment in a lake with the appropriate instrumentation and accuracy, or use established methodology to sample stream invertebrates (Arthurs, 2019; Scott et al., 2012). In addition to considering these three fundamental learning domains, UFEs may promote student outcomes that cross domains and/or enter the social realm, such as developing communication skills (Bell & Anscombe, 2013), building friendships and collaborations (Jolley et al., 2019; Stokes & Boyle, 2009), or developing a sense of belonging to a discipline or place (Kortz et al., 2020; Malm et al., 2020; O’Brien et al., 2020). Lastly, students participating in UFEs could result in broader, societal level outcomes, such as: students pursuing conservation efforts; contributing to citizen science projects; increased awareness of social justice issues; or support for sustainability efforts (Bell & Anscombe, 2013; Ginwright & Cammarota, 2015; Grimberg et al., 2008).

In Table 1, we present a list of common intended student outcomes from UFEs. The list of outcomes was propagated by UFE practitioners, first identified from a UFERN landscape study (O’Connell et al., 2020) and by participants at the 2018 UFERN meeting. O’Connell et al. (2020) surveyed practitioners on expected student outcomes from their UFEs. We then refined the list of outcomes by removing outcomes that were redundant, not measurable, or linked to very specific contexts (not field universal), and then grouped them by what we call “primary aim.” The primary aim category is an umbrella category by which to group similar intended outcomes. Table 1 illustrates a diversity of possible and likely outcomes from UFEs ranging across domains, but not every conceivable outcome is accounted for, and we encourage practitioners to consider outcomes that they do not see on this table if they are in alignment with their UFE. Interestingly, in O’Connell et al.’s (2020) survey of intended student outcomes in extended UFEs, the majority of respondents chose outcomes in the cognitive and/or psychomotor domains. Thus, students gaining content knowledge and skills is a prominent goal for practitioners of UFEs, but content can also be learned in many contexts. We and others propose that the distinctive impact of participation in a UFE may actually be more in the affective domain (Kortz et al., 2020; Van Der Hoeven Kraft et al., 2011). Thus, we encourage practitioners to consider focusing less on content‐level outcomes and more on the full spectrum of possible outcomes.

**TABLE 1:** Intended student outcomes and aligned assessment tool examples

Primary aim	Example student outcomes	Example assessment tools for measuring aim	Measurement details (# of items, item type, time to administer)	Population(s) tested	Ease of analysis	Original reference
Broader Relevance ‐ *development of awareness and connection beyond the context of the field experience*	Increased sense of connection to local/community problems or issues Increased sense of connection to large‐scale problems or issues Development as informed citizens	Perceived Cohesion Scale (PCS)	6 items, Likert	Multiple ages & populations	Easy	Bollen, K. A., and R. H. Hoyle. 1990. Perceived cohesion: a conceptual and empirical examination. Soc. Forces 69(2):479–504.
Connection to Place ‐ *relationships between people and the field environment*	Increased stewardship intention or behaviors Increased respect or care for the environment Stronger connections to place	Environmental Orientations (ECO)	16 items, Likert	Ages 6 – 13	Easy	Larson, L. R., Green, G. T., & Castleberry, S. B. (2011). Construction and Validation of an Instrument to Measure Environmental Orientations in a Diverse Group of Children. Environment and Behavior, 43(1), 72–89. https://doi.org/10.1177/0013916509345212
		Environmental Attitudes Inventory (EAI)	24 or 72 items, Likert	Multiple ages & populations	Easy	Milfont, T.L., and J. Duckitt. (2010). The environmental attitudes inventory: a valid and reliable measure to assess the structure of environmental attitudes. J. Envrion. Psychol. 30: 80–94.
		Place Attachment Inventory (PAI)	15 items, Likert	Multiple ages & populations	Easy	Williams, D.R., & Vaske, J.J. 2003, The measurement of place attachment: validity and generalizability of a psychometric approach: Forest Science, v. 49, p. 830–840.
		Place Meaning Questionnaire (PMQ)	30 items, Likert	Multiple ages & populations	Easy	Young, M., 1999, The social construction of tourist places: Australian Geographer, v. 30, p. 373–389, https://doi.org/10.1080/00049189993648.
		Place Meaning Scale‐Marine Environments (PMS‐ME)	34 items, Likert	Tourist industry representatives; resource managers; and recreational visitors	Easy	Wynveen, C. J., & Kyle, G. T. (2015). A place meaning scale for tropical marine settings. Environmental management, 55(1), 128–142.
		New Ecological Paradigm Scale (NEP)	15 items, Likert	Multiple ages & populations	Easy	Dunlap, R., K. Liere, A. Mertig, and R.E. Jones. 2000. Measuring endorsement of the new ecological paradigm: a revised NEP scale. J. Soc. Iss. 56: 425–442.
Nature of Science ‐ *Understanding of the process of science and how scientific knowledge is generated*	Increased awareness of scientific ethics Stronger sense of what life as a scientist is like Increased knowledge of the nature of science Increased proficiency in general research practices	Colorado learning attitudes about science survey ‐ biology (CLASS‐Bio)	31 items, Likert	Undergraduate students (University of Colorado and University of British Columbia)	Moderate	Semsar, K., Knight, J.K., Birol, G., and Smith, M.K. (2011). The Colorado Learning Attitudes about Science Survey (CLASS) for use in biology. CBE—Life Sciences Education, 10, 268–278.
		Views on the Nature of Science (VNOS‐C)	Open‐ended, 45–60 min	Multiple ages & populations	Hard (requires inter‐rater review of answers)	Lederman, N. G., F. Abd‐El‐Khalick, R. L. Bell, and R. S. Schwartz. 2002. Views of nature of science questionnaire: toward valid and meaningful assessment of learners’ conceptions of nature of science. J. Res. Sci. Teach. 39:497–521.
		Biological Experimental Design Concept Inventory (BEDCI)	14 items, multiple choice, 18 min	Undergraduate students (University of British Columbia)	Easy	Deane, T., K. Nomme, E. Jeffery, C. Pollock, and G. Birol. 2014. Development of the biological experimental design concept inventory (BEDCI). CBE–Life Sci. Educ. 13:540–551.
		Expanded Experimental Design Ability Test (E‐EDAT)	Open‐ended	Undergraduate students (University of Washington)	Moderate (Rubric)	S. E. Brownell, M.P. Wenderoth, R. Theobald, N. Okoroafor, M. Koval, S. Freeman, C. L. Walcher‐Chevillet, A.J. Crowe, How Students Think about Experimental Design: Novel Conceptions Revealed by in‐Class Activities, BioScience, Volume 64, Issue 2, February 2014, Pages 125–137, https://doi.org/10.1093/biosci/bit016
		Experimental Design Ability Test (EDAT)	Open‐ended, 10–12 minutes	Undergraduate students, Introductory class (Bowling Green State)	Moderate (Rubric)	Sirum, K., and J. Humburg. 2011. The experimental design ability test (EDAT). Bioscene J. Coll. Biol. Teach. 37:8–16
		The Rubric for Science Writing	Open ended	Undergraduates students and Graduate teaching assistants (University of Southern California)	Moderate (Rubric)	Timmerman, B. E C., D. C. Strickland, R.L. Johnson, and J. R. Payne. 2011. Development of a ‘universal’ rubric for assessing undergraduates’ scientific reasoning skills using scientific writing. Assess. Eval. Higher Educ. 36:509–547.
		Test of Scientific Literacy Skills (TOSLS)	Multiple Choice, 30 min	Multiple populations	Easy	Gormally, C., P. Brickman, and M. Lutz. 2012. Developing a test of scientific literacy skills (TOSLS): measuring undergraduates’ evaluation of scientific information and arguments. CBE–Life Sci. Educ. 11:364–377.
		Student perceptions about earth science survey (SPESS)	29 items, Likert	Undergraduate students in earth and ocean sciences (University of British Columbia)	Moderate	Jolley, A., Lane, E., Kennedy, B., and Frappé‐Sénéclauze, T. 2012. SPESS: a new instrument for measuring student perceptions in earth and ocean science. Journal of Geoscience Education, 60(1):83–91.
		Entering Research Learning Assessment (ERLA)	53 items, with 47 item optional paired assessment for mentors to assess trainee gains	Multiple populations of undergraduate and graduate trainees	Moderate (scoring guide)	Butz, A. R., & Branchaw, J. L. (2020). Entering Research Learning Assessment (ERLA): Validity evidence for an instrument to measure undergraduate and graduate research trainee development. CBE – Life Sciences Education, 19(2) https://doi.org/10.1187/cbe.19‐07‐0146
		Views about Science Survey (VASS)	30 items, Likert	8th‐undergraduate students	Easy	Halloun, Ibrahim. (2001). Student Views about Science: A Comparative Survey. Beirut: Phoenix Series/Educational Research Center, Lebanese University.
Personal Gains ‐*cognitive (e*.*g*., *content knowledge)*, *behavioral (e*.*g*., *skills)*, *and affective characteristics (e*.*g*., *comfort*, *confidence*, *self*‐*efficacy) gained through field experience*	Ability to live and work in primitive or adverse camping conditions Development of or increased “Grit” (perseverance through tough situation) Increased content knowledge Increased interest in the topic of field course More refined career goals Improved discipline‐specific skills Development of outdoor skills Increased confidence in physical fitness	Grit Scale (GRIT)	8 or 12 items, Likert	Multiple populations	Easy	Duckworth, A. L., Peterson, C., Matthews, M. D., & Kelly, D. R. (2007). Grit: Perseverance and passion for long‐term goals. Journal of Personality and Social Psychology, 92(6), 1087–1101.
		Climate change concept inventory	21 items, Likert	Undergraduate students	Easy	Libarkin, J. C., Gold, A. U., Harris, S. E., McNeal, K. S., & Bowles, R. P. (2018). A new, valid measure of climate change understanding: associations with risk perception. Climatic Change, 150(3–4), 403–416.
		Geoscience concept inventory (GCI)	select 15 question subset from 73 total questions, Multiple choice	Undergraduate students	Easy	Libarkin, J.C., Anderson, S.W., (2006). The Geoscience Concept Inventory: Application of Rasch Analysis to Concept Inventory Development in Higher Education: in Applications of Rasch Measurement in Science Education, ed. X. Liu and W. Boone: JAM Publishers, p. 45–73
		National Survey of Student Engagement (NSSE)*	70 items, Likert	Multiple populations	Easy	Kuh, G. D. 2009. The national survey of student engagement: conceptual and empirical foundations. New Direct. Inst. Res. 2009:5–20.
		Landscape identification and formation timescales (LIFT)	12 items, Multiple choice	Undergraduate students in earth and ocean sciences (University of British Columbia)	Easy	Jolley, A., Jones, F., and Harris, S. 2013. Measuring student knowledge of landscapes and their formation timespans. Journal of Geoscience Education, 61(2):240–251.
		Psychological Sense of School Membership (Class Belonging/School Belonging)	18 items, Likert	Middle school and undergraduate students	Easy	Goodenow, C. (1993). The psychological sense of school membership among adolescents: Scale development and educational correlates. Psychology in the Schools, 30, 79–90.
Personal Connections to Science Context ‐*affective characteristics such as comfort*, *confidence*, *self*‐*efficacy in science more broadly*	Greater sense of belonging in the scientific community Increased value for the interdisciplinary nature of science Increased interest in a general science career Increased interest in a field‐based science career Increased scientific self‐efficacy	Common Instrument Suite (CIS)*	10 items, Likert	Grades 4 and above	Easy	https://www.thepearinstitute.org/common‐instrument‐suite
		Motivated strategies for learning questionnaire (MSLQ)	81 statements, Likert		Easy	Pintrich, R. R., & DeGroot, E. V. (1990). Motivational and self‐regulated learning components of classroom academic performance, Journal of Educational Psychology, 82, 33–40.
		Science Interest Survey (SIS)	21 items, Likert	Middle and high school grade children from varying ethnic backgrounds	Easy	Lamb, R.L., Annetta, L., Meldrum, J. et al. MEASURING SCIENCE INTEREST: RASCH VALIDATION OF THE SCIENCE INTEREST SURVEY. Int J of Sci and Math Educ 10, 643–668 (2012). https://doi.org/10.1007/s10763‐011‐9314‐z
		Career Decision Making Survey ‐ Self Authorship (CDMS‐SA)	18 items, Likert	Multiple populations	Easy	Creamer, E. G., M. B. Magolda, and J. Yue. 2010. Preliminary evidence of the reliability and validity of a quantitative measure of self‐authorship. J. Coll. Student Devt. 51:550–562
		Research on the Integrated Science Curriculum (RISC)	Likert, adaptable		Easy	https://www.grinnell.edu/academics/centers‐programs/ctla/assessment/risc
		Student Assessment of Learning Gains (SALG)	5 item, Likert	College students (CSU‐Fullerton)	Easy	Student Perspectives on Curricular Change: Lessons from an Undergraduate Lower‐Division Biology Core Merri Lynn Casem. CBE—Life Sciences Education 2006 5:1, 65–75
		Science Motivation Questionnaire II (SMQII)	25 item, Likert	College students (University of Georgia)	Easy	Glynn, S. M., P. Brickman, N. Armstrong, and G. Taasoobshirazi. 2011. Science motivation questionnaire II: validation with science majors and non‐science majors. J. Res. Sci. Teach. 48:1159–1176.
		Survey of Undergraduate Research Experiences (SURE)	15 minute, Likert		Easy	Lopatto, D. 2004. Survey of undergraduate research experiences (SURE): first findings. Cell Biol. Educ. 3:270–277.
		Undergraduate Student Self‐Assessment Instrument (URSSA)	Likert, adaptable	Multiple undergraduates ‐ geared toward URE but mostly applicable	Easy	The Undergraduate Research Student Self‐Assessment (URSSA): Validation for Use in Program Evaluation Timothy J. Weston and Sandra L. Laursen CBE—Life Sciences Education 2015 14:3
		STEM Self‐efficacy (STEM‐SE)	29 items including demographic questions, Likert	Undergraduate students but with emphasis on historically underrepresented racial/ethnic groups in science majors engaged in research experiences	Easy	Byars‐Winston A, Rogers J, Branchaw J, Pribbenow, Hanke R, Pfund C. (2016). New measures assessing predictors of academic persistence for historically underrepresented racial/ethnic undergraduates in science. CBE–Life Sciences Education, 3ar32.
		STEM Career Interest Survey (STEM‐CIS)	44 items, Likert	Middle school students (grades 6–8) who primarily were in rural, high‐poverty districts in the southeastern USA	Easy	Kier M, Blanchard M, Osborne J, Albert J. (2014). The development of the STEM career interest survey (STEM‐CIS). Research in Science Education 44:461–481.
Transferable Skills ‐ *skills that can be applied to contexts outside of science*	Improved communication skills Improved collaboration skills Improved problem‐solving skills Improved critical thinking skills	Critical Thinking Assessment Test (CAT)*	15 items, Open‐ended	Multiple populations	Moderate (scoring guide)	Stein, B., A. Haynes, M. Redding, T. Ennis, and M. Cecil. (2007). Assessing critical thinking in STEM and beyond, p 79–82. In: Innovations in e‐learning, instruction technology, assessment, and engineering education. Springer, Netherlands
		California Critical Thinking Skills Test (CCTST)*	45 minutes, Multiple choice	Undergraduate students (CSU‐Fullerton)	Easy	Facione, P. A. 1991. Using the California Critical Thinking Skills Test in Research, Evaluation, and Assessment. [Online.]
		Self‐perceived communication competence (SPCC)	12 items, Numerical rating on 100 point scale	Undergraduate students	Easy	McCroskey, J.C., & McCroskey, L. L. (1988). Self‐report as an approach to measuring communication competence. Communication Research Reports, 5(2), 108–113.

Note

The intended student outcomes were first identified from the UFERN landscape study (O’Connell et al., 2020) and by participants at the 2018 UFERN Network Meeting at Kellogg Biological Station, April 30–May 2, 2018. The authors of this essay then refined the list by removing those outcomes that were either duplicated, irrelevant, not measurable, or linked to very specific contexts (not field universal). Each outcome is grouped according to a primary aim defined in the table below. The table organizes published assessment tools that fall under each primary aim category and that are applicable for use in undergraduate field education experiences. This table was designed to help practitioners identify instruments that align with the intended student outcomes they have identified for their field experiences. The primary aims are categories that the authors have defined to link outcomes with assessments using language that is accessible to the practitioner. The aim categories do not necessarily represent specific constructs or scales for individual assessments. The structure of the table follows that designed by Shortlidge and Brownell (2016).

#### 1.4.2 Consider the context of the UFE

UFEs can be highly variable in format (Lonergan & Andresen, 1988; O’Connell et al., 2020; Whitmeyer et al., 2009b). For example, some are strictly disciplinary (Jolley, Brogt, et al., 2018), others interdisciplinary (Alagona & Simon, 2010); they might occur locally (Peacock et al., 2018), in short duration (Hughes, 2016), over an entire course (Thomas & Roberts, 2009), or as a summer research experience held at a residential field station (Hodder, 2009, Wilson et al., 2018). O’Connell et al. (2021) comprehensively describe and organize the evidence for how student factors such as student identity, prior knowledge, and prior experience and design factors such as setting and social interaction influence learning in the variety of UFE formats (O’Connell et al., 2021). In this paper, we urge practitioners to consider student factors (e.g., prior knowledge, skills and experiences, motivation and expectations, social identity, and personal needs) and design factors (e.g., setting, timing, instructional models, and activities) when determining an appropriate assessment approach. These contextual factors should inform assessment decisions as well as data interpretation, and how to use the data to make decisions about next steps in assessment or evaluation. The intention is for practitioners to use the guide (Figure 1) to inform iterative change and improvement and reflective practice, not as static scaffolding.

### 1.5 Student factors

As with any learning environment, it is critical for instructors and staff to have a good idea of who the participating students are, and preempt what information may be pertinent to their experiences as practitioners plan to understand the outcomes of a UFE (Fakayode et al., 2014; Ireland et al., 2018; Pender et al., 2010; Stokes et al., 2019). In this way, student factors may influence the selection of appropriate assessment approaches and tools. There are a number of factors that can be considered when designing and understanding the outcomes of assessment; here, we provide numerous examples for contemplation.

For example, a factor to consider is prior student knowledge and skills. Imagine two UFEs: In the first UFE, students are upper‐division physiology majors studying endemic amphibians’ responses to changes in stream water quality; the second UFE is designed for non‐science majors to broadly survey the biodiversity of local flora and fauna. If a practitioner decides they want to identify whether/how students’ attitudes change regarding the local environment as a result of the UFEs, they might select a survey designed to collect data on environmental attitudes (e.g., Table 1, Primary Aim: Connection to Place; Assessment Tool: Environmental Attitudes Inventory (EAI), Milfont & Duckitt, 2010). The physiology students from the first example may begin the UFE with largely positive environmental attitudes already. Thus, administering a survey at the beginning and end of the UFE (pre–post) to measure this construct may not reveal any gains. Yet, in the second UFE example, the students are introductory, non‐science majors, and they may demonstrate significant, quantifiable gains in environmental attitudes. Therefore, in the physiology student example, this specific outcome was not detectable due to a measurement limitation called the ceiling effect. This effect can occur when a large proportion of subjects begin a study with very high scores on the measured variable(s), such that participation in an educational experience yields no significant gains among these learners (Austin & Brunner, 2003; Judson, 2012). In this case, instead of the survey, the practitioner might learn more by crafting an essay assignment that probes the physiology students’ environmental values. This option would demonstrate consideration of the student population in the assessment strategy.

Other factors to consider might include student motivation and expectations. An assessment of students in a pair of geoscience UFEs in New Zealand showed that study abroad students were more intrinsically motivated, pro‐environmental, and had a stronger sense of place than local students in a similar field experience, although they were held in the same place (Jolley, Kennedy, et al., 2018). This assessment highlighted the need to adapt the design of the field experience to be more applied, environmentally focused, and place‐based, rather than simply applying the same curricula unchanged to a different student population (Jolley, Kennedy, et al., 2018). Here, future assessments could be targeted toward investigating whether the revised UFE design for study abroad students effectively captured their motivation and interest. And/or, a deeper qualitative investigation could be conducted to characterize their field experiences in relation to the environmental and place‐based content.

Prior experiences and identity are also critical to consider (Morales et al., 2020; Scott et al., 2019). Have the students experienced fieldwork already? Practitioners might want to know what proportion of the students are first‐generation college students, or if students have prior conceptions of fieldwork. Such knowledge could guide an assessment approach aimed at understanding how first‐generation students experience the UFE compared with continuing generation students; or in the latter case, if students hold accurate or inaccurate conceptions (or any conception at all) about fieldwork.

Also important is awareness of personal needs such as safety and well‐being, especially for students of often marginalized identities such as BIPOC (Black, Indigenous, and People of Color) students and LGBTQ + students (Anadu et al., 2020; Demery & Pipkin, 2021; Giles et al., 2020; John & Khan, 2018; Marín‐Spiotta et al., 2020). These considerations can influence the implementation of an assessment strategy, as participants will experience different levels of comfort and risk based on the questions being asked. Students may be less comfortable sharing if they already have concerns about safety in the field environment and culture of UFEs. Even on an anonymous survey, students may be worried about being personally identifiable if they are one of few students of a particular identity or combination of identities. Ensure that students are provided full and complete information about what will be done with their data, have the opportunity to ask questions, and are free from coercion. In some cases, this may mean having someone who is not the course instructor conduct the assessment. Although questions like these would be addressed if the study requires approval through an IRB or similar, we encourage their consideration regardless as they have a bearing on student comfort and perceptions of safety.

Programmatic processes such as recruitment efforts or selection criteria can also influence student factors (e.g., O’Connell et al., 2021; Zavaleta et al., 2020). Are all students enrolled in a class participating in the UFE (as in a CURE), do they self‐select, or are they chosen to participate based on certain criteria? It is important to keep in mind that any outcomes from a UFE are only representative of the students who actually participated, and thus not broadly representative of any student who might participate. In summary, when applying the assessment strategy presented in this paper, one must consider the following: Are the UFE outcomes reasonable to achieve and measure given the specific student population? Student factors must be considered in UFE design and will likely moderate or even become the subject of assessment efforts.

In the vignettes, we identify various factors that may inform program design/UFEs and provide diverse examples in which the assessment approaches are aligned with the student population. For example, some programs specifically engage students with a background or interest in STEM (e.g., Figure 2a, b), and others are open to all majors (e.g., Figure 2c).

### 1.6 Setting and timing

Fundamental to the definition of UFEs is that they are immersive, communal, and somewhat unstructured (even if conducted remotely) (Posselt, 2020, p. 56–57). This distinctive learning environment should be considered when picking an assessment approach and interpreting assessment data. If a practitioner wanted to evaluate how a UFE impacts student knowledge of a particular concept, then a two‐week, on‐campus UFE focused on urban greenspaces may yield less deep learning about forest ecology than a semester‐long field course held in a live‐in forest field station. Thus, a summative assessment on forest ecology concepts should be reflective of the amount of time and depth the students have had to amass relevant cognitive gains.

Previous work indicates that instructors and students place high value on UFEs where participants live and work together in the field (Jolley et al., 2019). However, cohabitation and isolation may also present challenges in the way of mental health stressors (John & Khan, 2018) and unfamiliar and overstimulating environments (Kingsbury et al., 2020). In an almost opposite, yet timely and relevant example, Barton (2020) describes how remote UFEs need to reduce or change expected learning outcomes specific to being “in the field” to outcomes more relevant. Considering how the UFE setting might impact student learning should be factored into determining intended student outcomes, and subsequently how to test whether those outcomes are being met. Figure 2 illustrates how factors such as residential/non‐residential settings, length of the UFE, and accessibility of the setting can inform assessment strategies.

### 1.7 Contextual factors can intersect

The student experience (and thus the student outcomes) are influenced by the intersection of setting and timing factors, making interpretation of the results complex. For example, perhaps a student is a primary caregiver for someone at home and is distracted by irregular or absent cellular service, therefore are unable to establish a connection to place due to distraction and worry. Some students may identify that eating as a community helps them to establish a sense of belonging among peers and instructors, whereas eating in a group setting may cause a student with a complex relationship with food to experience extreme discomfort. These examples are provided to highlight how residential or community settings may have contradictory impacts on different students in the same UFE; thus, it may not always be appropriate or meaningful to solely look at assessment findings on an average or “whole‐class” scale.

### 1.8 Instructional model and activities

As with any learning experience, working backwards from the specific learning outcomes will help instructors to ascertain whether the curriculum is in alignment with those goals, or whether there are activities that are not aligned or extraneous. If intended student outcomes are to increase skills with research practices (e.g., Figure 2A), then the actual activities should support this outcome. In this vignette, students are supported to develop a research project, aligning the instructional model and activities to the outcome. Similarly, an intended outcome of the *Humanities Course at a Field Station* vignette (Figure 2c) was to develop stronger connections to place in Northern Michigan, and the course curriculum included activities focused on exposure to place, and fostering a sense of place. In the *Urban Field CURE* vignette (Figure 2b), an intended outcome was for students to engage with relevant stakeholders, accordingly, the students received feedback on thier experimental design from the stakeholders. There are multiple options for designing curriculum or activities that will allow practitioners to gauge the participant experience, thus acting as a form of formative assessment. For example, designing a written reflection activity that probes the student experience or their learning in that particular environment, or collecting student artifacts from the UFE can yield information regarding how a student experiences the UFE, and can in turn inform UFE stakeholders.

### 1.9 Accessibility and inclusion

As illustrated previously, basic characteristics of the location and pedagogy of the UFE can have an impact on the physical, cognitive, and/or emotional accessibility of the learning environment for various students. In efforts to include as many students as possible, it is important to consider factors such as physical space (e.g., restroom availability, non‐gendered housing, housing for students with physical, emotional, or psychological concerns), quality of Internet connection (if remote), sleeping arrangements, skills needed to participate (e.g., training in swimming), or other health concerns (e.g., allergies). Additionally, social isolation/inclusion can be especially prevalent in UFEs for students who do not share the same identities with other participants and/or are from underrepresented groups (Atchison et al., 2019; Morales et al., 2020). *One of the vignettes* (Figure 2d) is specifically tied to accessibility and demonstrates the importance of directly working with students and faculty with disabilities on a field trip in order to address the intended outcomes of the UFE.

#### 1.9.1 Assessment approach

Key to choosing an assessment approach is first asking: What is the motivation for collecting the data? As discussed earlier, there are a number of reasons and ways one might assess a UFE including: identifying if students are meeting specific learning goals; to collect publishable data on students’ sustained interest in a topic; or to identify if the UFE is meeting programmatic goals to report back to a funding agency or university. Regardless of stakeholders’ motivations, using backward design to clarify and align program goals, activities and assessments will allow for a solid platform for improvement and evaluation.

We recommend that practitioners consider both formative and summative assessments. A formative assessment might be a UFE student completing a written reflection or keeping a “reflective diary” (Maskall & Stokes, 2008; Scott et al., 2019) regarding an aspect of their learning experience. This strategy would provide students a chance to reflect on their learning process and their changing experience and competencies in their own words. Further, such a formative assessment would allow instructors/stakeholders to better understand how programming, or more specifically a particular aspect of programming may impact student perceptions and possibly how to adjust the learning experience. A summative assessment strategy could be employed if practitioners wanted to know whether students have gained a greater appreciation for the natural world as a result of a UFE, which could be measured for example by conducting a pre/postsurvey designed to measure this specific construct (e.g., Table 1. Primary Aim: Connection to Place, Assessment Tool: Place Attachment Inventory (PAI), Williams & Vaske, 2003). Figure 1 is meant to be useful in planning assessment strategies but could also serve as a helpful communication tool when engaging with funders and stakeholders.

It may also be appropriate to hire an external evaluator. An advantage of external evaluation is that it presumably provides an unbiased view of the program, as the evaluator will assess the impacts of programming on participants and report findings in an objective manner. From the evaluator's perspective, is the program meeting its intended goals? For whom does the UFE appear to be “working,” and are there certain student groups that are not being impacted in the way designers of the experience had intended? An external evaluator will often work with the team to identify goals and then conduct a holistic programmatic evaluation, including all stakeholders. The caveat regarding external evaluation is cost. If grant‐funded, external evaluation may be encouraged or even required; if not grant‐funded, finding funding would be necessary in order to hire the evaluator or evaluation team.

### 1.10 Data collection and analysis

Deciding what type of data to collect will require having a reasonable idea of the program's goals and anticipated outcomes, as well as an awareness of the time it will take to collect and analyze the type of data collected. Practitioners may consider using quantitative measures such as surveys, or qualitative methods such as interviews or open‐ended questions. A mixed methods approach can employ both qualitative and quantitative methodology, allowing for a more nuanced understanding (Creswell & Clark, 2007). Identifying if the intention is to publish the data (requiring IRB review), or to use it internally to gain a better understanding of an aspect of programming should play a key role in determining the approach and the “rigor” with which one collects and interprets the data.

Using best practices in research will help to avoid conflicts of interest, and better ensure that valid and reliable data are collected (Ryan et al., 2009). If, for example, a program recruits students for interviews after they participate in a UFE, someone outside of the UFE leadership or instructional team should be the interviewer. This practice would minimize the power differential between participant and researcher, thereby ensuring that UFE interview participants feel that they can be honest about their experiences, and not worry about pleasing or offending those involved in the program (Kvale & Brinkman, 2009). Further, the interview questions should be vetted by others (similar to the target population) before the interviews begin to ensure that the questions are interpreted by the participants as they are intended.

Using appropriate methodology in planning data collection and conducting analyses, will allow for apt interpretation of the results (Clift & Brady, 2005). As illustrated in the vignettes (Figure 2d), deeply understanding the lived experiences of participants may call for knowledge of qualitative methodology. One may not want to conduct numerous interviews with students and staff without the resources to hire researchers, or ample time to analyze the data. Analyzing rich qualitative data typically involves iterative “coding” by multiple trained researchers who develop and revise codebooks and then apply those codes to the transcribed text, regularly checking for coding reliability among researchers (Belotto, 2018; O’Connor & Joffe, 2020; Saldaña, 2011). Coding processes can vary, sometimes guided by a theoretical framework, a priori ideas, and/or they may allow for inductive, deductive, or a combination of coding approaches (see Saldaña, 2015 for a comprehensive manual on coding).

Similar to qualitative data, quantitative data collection and analysis requires planning and expertise. Researchers will want to ensure that the research aims are well‐aligned with the data collection methods or tools, and in turn, allow for appropriate interpretation the data. Comparing pre–post survey responses would be one seemingly straightforward way to measure change over time in participant learning (e.g., Figure 2C). Yet, we do caution against simply pulling a tool from Table 1 or elsewhere and simply assuming that by using it, it “worked.” We recommend collaborating with experts who are familiar with quantitative methods. Using a survey tool may yield quickly quantifiable results, but if the survey has not undergone vetting with individuals similar to the population of study, or it has not previously shown to collect valid data in very similar populations, one cannot assume that the data collected are valid or reliable (Barbera & VandenPlas, 2011; Fink & Litwin, 1995). Just as we do not use micropipettes to measure large volumes of lake water, we would not use a tool developed to measure academic motivation in suburban elementary school students to measure motivation of college students participating in a residential UFE and expect to trust the survey results outright. If a tool seems appropriate for a given UFE and the student population, we encourage first testing the tool in that population and work to interpret the results using best practices (for a comprehensive resource on these practices, see American Educational Research Association (AERA) 2014). As described previously, Table 1 consists of several assessment tools which are potentially relevant for measuring UFE outcomes. We only included tools that have been peer‐reviewed and published in the table. We strongly recommend reviewing the associated peer‐reviewed paper before using a tool, as well as looking in the literature to see whether others have used the tool and published their findings.

It is also possible that one would want to measure an outcome for which a tool has not yet been developed. In this case, working on an attuned assessment strategy based on iterative adaptations and using lessons learned may be appropriate (Adams & Wieman, 2011). There are many steps involved with designing and testing a new assessment tool that is capable of collecting valid and reliable data. Therefore, if stakeholders deem it necessary to create a new tool to measure a particular outcome, or develop or modify theory based on an UFE, we recommend working with psychometricians or education researchers.

#### 1.10.1 What are the next steps?

We encourage that the process of evaluation and assessment is a reflective, cyclical, iterative process of improvement as it relates to UFE design and implementation. There are inevitably going to be aspects of any learning experience that could be improved, and this guide to assessment (Figure 1) can help practitioners visualize alignment between intended outcomes, programming, assessment, and evaluation; and how each informs the other. The next steps for many UFEs might be to first report to stakeholders (funders, the institution, etc.) on the outcomes of the UFE. Or, if the goal of the assessment effort was to conduct novel research, then the next steps might be to analyze, write up, and submit the results of the study for peer review, thereby contributing to the growing literature of empirical outcomes from UFEs. For example, one vignette (Figure 2b) describes how the assessment strategy will provide pilot data for ongoing publishable projects. Other vignettes (Figure 2a,c) illustrate how results from assessment efforts can be leveraged to apply for or validate grant funding. These types of data may be paramount to sustained funding, data‐driven advocacy efforts, and/or applying for future funding for continued programming.

An important part of the presented strategy is that it might be used to engage stakeholders in a discussion about what additional questions might be appropriate to ask or what improvements need to be considered. Is there alignment between activities and learning goals? Is the current evaluation strategy accurately measuring what stakeholders expect the students to gain from the UFE? Is the programing intentionally inclusive of the participants’ diverse perspectives and experiences, or could adaptations be made to better serve the UFE population? For example, to address financial and relocation barriers identified through the program evaluation for one field‐based REU, the REU leaders introduced new policies for students to be paid at the start of their experience and identified field research projects that were located in student communities, and in another case, accommodations were made for the student's family to join them as part of the residential field experience (Ward et al., 2018). This is just one example of how assessment data can be used to inform the design of future UFEs and highlights how the assessment process can be both informative and iterative.

## 2 EXPANDED VIGNETTES

Here, we provide detailed narratives that more fully illustrate two of the vignettes introduced in Figure 2 (Figure 2a,c). The expanded vignettes are intended to transform the collective ideas presented here and summarized in Figure 1 into concrete examples, serving as an example to guide assessment of diverse UFEs.

### 2.1 Vignette a—Summer research experience for undergraduate students (Figure 2a)

#### 2.1.1 The field site and course

The Thomas More University (TMU) Biology Field Station was founded in 1967 and offers research, courses, and field experience programs for undergraduate students and outreach programs for K‐12 students and the general public. The TMU Biology Field Station is located 20 miles from the main campus in a more remote/unpopulated setting, along the banks of the Ohio River. Each summer, undergraduate students from around the country are selected to participate in a 10‐week summer research internship where they are assigned to one of three long‐standing research projects and develop an independent‐study side project on which to develop and work throughout the ten weeks.

#### 2.1.2 Development of student outcomes

During the preceding academic year, TMU Biology Field Station staff, including the field station director, discussed outcomes that they wanted to achieve with these internships. These outcomes were informed by discussions with the faculty from the Department of Biological Sciences at TMU and with collaborating researchers at the Environmental Protection Agency (EPA) Office of Research and Development and the US Fish and Wildlife Services (USFW). The primary, intended student outcomes included (1) increased understanding of and proficiency with research practices and processes; (2) increased understanding of discipline‐specific concepts and content; and (3) stronger skills in discipline‐specific methods and procedures. Secondary student outcomes included (1) expanded professional networks; (2) greater sense of belonging in the scientific community; (3) more refined career goals; and (4) stronger professional skills.

#### 2.1.3 Course and field station context

To qualify, students must have completed one year of general biology and/or one year of general chemistry while maintaining a 3.0 minimum GPA. The qualifications to apply are kept at a minimum, by design, to ensure that first‐year students are eligible to apply. No prior research experience was required. The application process was open in December; applications were due in early February; and selections were made in early March for the subsequent summer. Phone or face‐to‐face interviews were conducted with each finalist as part of the application process. All interns were required to live on site. A stipend and free housing were provided.

During the internship, students were assigned to one of three long‐term projects at the TMU Biology Field Station and conducted this research as part of a small group of students and one faculty mentor. In addition, students were required to conduct a small‐scale independent‐study project of their own choosing, in collaboration with a faculty mentor. For the independent‐study project, students were required to conduct a literature search, write a proposal, and carry out the project within the course of their summer internship. At the conclusion of the summer, students made on oral presentation on their group work and a poster presentation on their independent project.

In addition, student interns were required to attend a summer seminar series during which professionals presented their research and spent a day observing the students in action. Lastly, students participated in field trips and tours to laboratories at the EPA, USFW, and local governmental agencies and served as mentors for a weeklong STEM camp for high school students.

The TMU Biology Field Station is a residential field station, where students live together in houses. In addition to the residential structures, there are three laboratories, four classrooms, and a STEM Outreach Center. Students, staff, and faculty eat meals together and socialize together in both formal and informal activities throughout the summer.

#### 2.1.4 Data collection

In order to assess change (increases in perceived ability or value), the field station director used a pre/postsurvey to identify student perceptions before they began the internship and after they ended the internship. The survey included measures about research practices and processes, discipline‐specific concepts and content, and discipline‐specific methods and procedures. The survey also included measures about career goals and professional skills. The field station director also conducted mid‐summer and exit interviews with each student intern to explore perceptions about their knowledge and skills gained through the program. While this assessment was created for an institutional annual report, the Director also used these data for support of additional external funding in grant applications and also compared the findings to previous years’ surveys.

#### 2.1.5 Next steps

Findings from the survey responses and interviews indicated that students in the internship program gained knowledge and skills in research practices and in discipline‐specific content, methods, and procedures. Further, students indicated more refined career goals and professional skills, namely oral and written skills. Students in the internship perceived increased confidence in their ability to communicate about science and an increased scientific network.

Future assessment work will consist of additional surveys and interviews with students a year later to explore how the internship experience impacted their academic work in the subsequent school year and career development. Lastly, attempts are being made to contact student interns from previous years to determine their specific career path and status.

### 2.2 Vignette c—Humanities course at a field station (Figure 2c)

#### 2.2.1 The field site and course

University of Michigan Biological Station (UMBS), which was founded in 1909, houses research, courses, and field experience programs for students. UMBS is located 250 miles from central campus in a remote setting. The *Humanities Course at a Field Station* was a newly designed course which was part of a larger effort to bring students from other disciplines to UMBS.

#### 2.2.2 Development of student outcomes

During the humanities course development, UMBS staff, including the program manager and program evaluation coordinator, discussed outcomes that they wanted to explore with this particular class to include in their annual program assessment. These outcomes were informed by discussions with the faculty as well as through reviewing syllabi. The intended student outcomes included (1) develop stronger connections to place in northern Michigan; (2) increased ability to communicate about scientific work; and (3) increased value for the interdisciplinary nature of science.

#### 2.2.3 Course and field station context

The humanities course was open to all undergraduate students across majors, room and board was free for the first year of the program for students, scholarship assistance was available, and transportation was provided. The course ran for six weeks during the UM spring term, which allowed students opportunities to work or take other courses during the rest of the summer. The course was a place‐based course, where the focus was on learning from the place and not just about the place. Students involved in this course took four short courses and received 8 credit hours across three departments (English, Anthropology, and American Culture); each course was taught by a different instructor.

UMBS is a residential field station, where students live together in cabins and faculty also live on site. Students and faculty eat meals together in the dining hall. Five other undergraduate courses ran at the same time as the humanities course. These additional five courses came from more traditional biophysical disciplines such as general ecology and biology of birds. While students in the humanities course generally spent time with their classmates and faculty in their individual course, there were opportunities (both structured and unstructured) for students to communicate, work with, and form connections with students, researchers, and faculty in other courses.

#### 2.2.4 Data collection

In order to assess change (increases in perceived ability or value), the program evaluation coordinator used a pre/postsurvey to identify student perceptions before they began the course and after they ended the course. The survey included measures about sense of place, sense of connection to larger‐scale problems or issues, and ability to communicate with scientists about scientific work. The program evaluation coordinator also conducted a focus group with students in the course to explore perceptions about their value of the interdisciplinary nature of science, ability to communicate, and connections to place in more detail. Interviews with the instructor and a focus group with the TA for the course also provided insight into change in student perceptions about these topics and *how* these changes developed in their time taking this course at UMBS.

While this assessment was created to share for an annual report, the program evaluation coordinator was interested in sharing this information with the larger field education community, and so all of the assessment of this course (and all courses at UMBS) had IRB approval. In addition, the program evaluation coordinator selected published measures to include on pre/postsurveys that had been tested in college populations. The program evaluation coordinator intentionally conducted focus groups because students had no interaction with her until this meeting and she was not associated with their grades or evaluation for their course.

#### 2.2.5 Next steps

Findings from the first year of survey responses and focus groups indicated that students in the course formed extremely close‐knit bonds. Future assessment work will consist of interviews with students, faculty, and TA to explore how connections to others (sense of belonging in the class) impact learning and understanding of different course topics.

In addition, findings from surveys and focus groups indicated that students in the course perceived increases in the value of the interdisciplinary nature of science and increased confidence in their ability to communicate about science. Findings from faculty interviews supported student responses and also indicated that faculty had a strong interest in doing more intentional collaboration with biophysical courses in the future. After discussing all of the assessment data, UMBS staff decided to expand their assessment for the next year. Specifically, they wanted to know whether students from biophysical courses who interacted with students in the humanities course also experienced increases in perceived value of the interdisciplinary nature of science and ability to communicate about science. The program evaluation coordinator intends to add additional assessment approaches to examine interactions between this course and other courses at the station. This may include observations of structured and unstructured activities with the humanities and biophysical courses as well as adding survey questions and/or focus group questions for all students who are taking courses at UMBS. Thus, the results of the assessment of the humanities course not only addressed whether the student outcomes were achieved in the humanities course, but also highlighted changes in the program that would happen in future iterations, and informed additional assessment of all UMBS courses in the next year.

## 3 CONCLUSIONS

We encourage using contextual information about a UFE to iteratively inform assessment strategies and in turn ‐ improve the value and inclusivity of the UFE for the full spectrum of participants and stakeholders. We encourage practitioners to use the supports provided here to conduct applied research aiming to understand how various characteristics of UFEs impact various student populations, essentially to “identify what works for whom and under what conditions.” (Dolan, 2015; National Academies of Sciences and Medicine (NASEM), 2017) p. 175). In general, we have little empirical evidence about the linkage of program characteristics to learning outcomes in UFEs. O’Connell et al. (2021) present an evidence‐based model that hypothesizes how *student context factors* and *program design factors* (or program characteristics) impact student outcomes in UFEs. Through a thoughtful assessment approach along with consideration of student context factors, practitioners may begin to unravel which design factors of their UFE are specifically leading to which student outcomes for which students. Future work could model which design factors lead to specific outcomes, as demonstrated by work to better understand how CURE elements influence student outcomes (Corwin et al., 2015).

We believe that the process of informed assessment and reflection will improve the accessibility and inclusivity of UFEs. Morales et al. (2020, p. 7) call for continuing a “conversation about creating student‐centered field experiences that represent positive and formative experiences for all participants while removing real or imagined barriers to any student participating in field research.” Explicit attention to diversity, equity, access, and inclusion regarding who gets to participate in UFEs and the learning that results from the experiences are key conversations with important implications (Carabajal et al., 2017; Demery & Pipkin, 2021; Giles et al., 2020; Morales et al., 2020; Nairn, 1999; Stokes et al., 2019; Zavaleta et al., 2020). As illustrated in Figure 2d, for example, authentically considering what it means to be accessible and inclusive is an important question, and we suggest that practitioners begin to systematically evaluate who is served by their UFE and who is not served and why, thus deeply investigating how the UFE may become more inclusive for diverse individuals. It will be necessary to work across disciplines to learn what is needed to support and advocate for accessible and inclusive UFEs such that as many students as possible can participate and have a positive experience.

The recent COVID‐19 pandemic has brought to the forefront vital questions about the role of virtual field experiences (Arthurs, 2021; Swing et al., 2021), as well as aligned assessment practices. We suggest that this is one area where developing novel assessment tools is needed to effectively measure impact and to ask such questions as: What are the characteristics defining a virtual or remote UFE? As it relates to outcomes, what can we learn about the impacts of in‐person experiences vs. remote on a student's affect such as their sense of belonging?

Here, we meet a call from the community to aid practitioners and stakeholders in using best practices to assess, evaluate, and/or research the spectrum of UFEs. UFEs are widespread and diverse, yet unique and complex. As we consider more deeply the outcomes that are specific to UFEs, we urge practitioners to move toward evidence‐based advocacy and improvement for the continued support of UFEs.

## CONFLICT OF INTEREST

The authors declare no competing interests.

## AUTHOR CONTRIBUTION

**Erin E. Shortlidge:** Conceptualization (equal); Data curation (equal); Investigation (equal); Methodology (equal); Project administration (equal); Visualization (equal); Writing‐original draft (equal); Writing‐review & editing (equal). **Alison Jolley:** Conceptualization (equal); Data curation (equal); Investigation (equal); Methodology (equal); Visualization (equal); Writing‐original draft (equal); Writing‐review & editing (equal). **Stephanie Shaulskiy:** Conceptualization (equal); Data curation (equal); Investigation (equal); Methodology (equal); Visualization (equal); Writing‐original draft (equal); Writing‐review & editing (equal). **Emily Geraghty Ward:** Conceptualization (equal); Data curation (equal); Investigation (equal); Methodology (equal); Visualization (equal); Writing‐original draft (equal); Writing‐review & editing (equal). **Christopher N. Lorentz:** Conceptualization (equal); Data curation (equal); Investigation (equal); Methodology (equal); Visualization (equal); Writing‐original draft (equal); Writing‐review & editing (equal). **Kari O'Connell:** Conceptualization (equal); Data curation (equal); Funding acquisition (equal); Investigation (equal); Methodology (equal); Project administration (equal); Supervision (equal); Visualization (equal); Writing‐original draft (equal); Writing‐review & editing (equal).

## Data Availability

N/A.
